# Different Erythrocyte MicroRNA Profiles in Low- and High-Altitude Individuals

**DOI:** 10.3389/fphys.2018.01099

**Published:** 2018-08-14

**Authors:** Liping Sun, Fengyan Fan, Ruilin Li, Beifang Niu, Liguo Zhu, Shuai Yu, Shuying Wang, Cuiying Li, Deqing Wang

**Affiliations:** ^1^Department of Blood Transfusion, Chinese PLA General Hospital, Beijing, China; ^2^Department of Blood Transfusion, Air Force General Hospital, PLA, Beijing, China; ^3^Department of High Performance Computing Technology and Application Development, Computer Network Information Center, Chinese Academy of Sciences, Beijing, China; ^4^University of Chinese Academy of Sciences, Beijing, China; ^5^Guizhou University School of Medicine, Guiyang, China; ^6^Clinical Laboratory, Hainan Branch of PLA General Hospital, Hainan, China

**Keywords:** high-altitude hypoxia, Tibet, Han Chinese, Tibetan, erythrocyte-derived microRNAs

## Abstract

**Background:** The number of red blood cells (RBCs) increases significantly in response to high-altitude hypoxic environments, and the RBC microRNA (miRNA) expression pattern is similar to that in whole blood. Studies have shown that miRNA in plasma can act as a circulating hypoxia-associated marker, but the effect of a high-altitude hypoxic environment on RBC-derived miRNAs has not yet been reported.

**Methods:** Blood samples were collected from 20 Han Chinese individuals residing at 500 m (Sichuan Han), 10 migrant Han Chinese citizens residing at 3,658 m (Tibet Han) and 12 native Tibetans, and RBC indices measurements and miRNA sequencing analyses were performed for the three sample groups. The levels of some markedly altered miRNAs at high altitude were subsequently measured from 5 randomly selected samples of each group by real-time PCR. Bioinformatic analyses was performed to determine the potential target genes of selected hypoxia-associated miRNAs.

**Results:** Marked changes of several RBC indices were observed among the Tibet Han population, the Tibetan population and the Sichuan Han population. A total of 516 miRNAs derived from RBCs were initially identified by miRNA sequencing in the three sample groups. Compared with the Sichuan Han population, 49 miRNAs were differentially expressed in the Tibet Han population (17 upregulated and 32 downregulated). 12 upregulated and 21 downregulated miRNAs were observed in the Tibetan population compared with the Sichuan Han population. A total of 40 RBC miRNAs were differentially expressed in the Tibetan population (15 upregulated and 25 downregulated) compared with the Tibet Han population. Two significantly altered miRNAs with the highest expression levels (miRNA-144-5p and miR-30b-5p) were selected for real-time PCR analysis, and the results were consistent with those of miRNA sequencing. Furthermore, bioinformatic analyses showed that some potential target genes of miR-144-5p and miR-30b-5p are involved in the erythroid- hypoxia-, and nitric oxide (NO)-related signaling pathways in response to hypoxia.

**Conclusion:** Our findings provide clear evidence, for the first time, that a high-altitude hypoxic environment significantly affects human RBC miRNA profiles.

## Introduction

The most extreme environment that people face is the hypoxic environment of the Tibet Plateau. The Tibet Plateau is the highest plateau in the world with a mean altitude of over 4,500 m. People who live at high altitude for a long time are prone to high-altitude polycythemia, high-altitude heart disease, plateau depression and other chronic altitude diseases (Simonson et al., 2010; He et al., 2013; Huertasánchez et al., 2014). Studies have shown that genotype variation at several unique genes (e.g., EGLN1, PPARA, and EPAS1) provide genetic contribution to an individual's adaptation to high-altitude hypoxic environments (Simonson et al., 2010; Yi et al., 2010). However, the epigenetic basis of this adaptation remains unknown.

The current literature shows that hypoxia is an important proximal regulator of miRNA production and function and that miRNAs can rapidly respond to hypoxia-induced physiological changes in the cell (Yan et al., 2009; Nallamshetty et al., 2013; Rupaimoole et al., 2014). Recently, some reports have revealed that circulating miRNA concentrations play an important role in the response to hypoxia at high altitudes. Yan et al. showed that the miRNA expression of plasma and peripheral blood cells is significantly different in high-altitude hypoxic environments (Yan et al., 2015). Their follow-up work confirmed that the miR-210-3p level is significantly altered in the peripheral blood cells of a Tibet Han group compared with a Han group (Yan et al., 2016). Most recent research suggests that miRNA expression levels in the plasma are associated with the occurrence of acute mountain sickness (Liu et al., 2017). In a high-altitude hypoxic environment, the number of RBCs changes significantly to enable adaptation to hypoxia. However, the impact of high-altitude hypoxia on RBC-derived miRNAs remains unclear.

Several studies have reported that the whole blood miRNA expression pattern is similar to that of RBCs but not leukocytes (Chen et al., 2008; Kannan and Atreya, 2010; Wu et al., 2017). Chen et al. also showed that miRNA profiles in human reticulocytes were very different from those in mature RBCs, indicating that a diverse population of miRNAs are selectively retained in RBCs, as opposed to being a random remnant (Chen et al., 2008; Hamilton, 2010). Among the hundreds of RBC miRNAs, the most abundant are miR-451a, miR-144-3p, miR-16, miR-92a, let-7, miR-486-5p (Doss et al., 2015). miR-451 and miR-144 are upregulated by GATA and play crucial roles in erythroid development (Rasmussen et al., 2010). miR-16-5p, miR-451a-5p, miR-486-5p, and miR-92a-3p are the most abundant intracellular Ago2-bound miRNAs observed in 24-h-stored RBCs, and functional enrichment analysis of mRNA targets of these major miRNAs have identified molecules related to various diseases, biological functions, and toxicity functions (e.g., cardio-, hepato-, and nephrotoxicity) (Vu et al., 2017). Consequently, selective retention of active miRNAs and the existence of Ago2-miRNA in RBCs indicate that RBC-derived miRNAs likely have specific functions.

Interestingly, several observations provide direct evidence defining the important function of miRNAs in RBCs (Regev-Rudzki et al., 2013; Mantel et al., 2016; Wang et al., 2017). For instance, studies have shown that the Ago2-miR-451 complex derived from infected RBCs is taken up by endothelial cells and can alter vascular function through RBC extracellular vesicles in malaria, even though RBCs lack the target mRNAs of miRNAs inside the cells (Mantel et al., 2016). Therefore, more attention should be directed toward understanding RBC miRNAs. It has been reported that RBC miRNAs not only inhibit the reproduction of Plasmodium falciparum in patients with sickle cell anemia but are also associated with the development of bodily disorders (Lamonte et al., 2012; Duan et al., 2016, 2017; Walzer and Chi, 2017; Wu et al., 2017). In this study, we hypothesized that high-altitude hypoxic environments likely impact RBC miRNA expression levels.

To test our hypothesis, RBC indices of people residing in the low- and high-altitude areas were measured. miRNA sequencing screening and real-time PCR confirmation assays were performed to systematically and comprehensively evaluate and compare RBC miRNA profiles between Han Chinese residing in Sichuan (at 500 m) and Han Chinese who migrated from the plain area to Tibet (Tibet Han, at 3,658 m) as well as the ethnic Tibetan population.

## Methods

### Participants and RBC sample processing

A total of 42 blood samples were collected from 20 Han Chinese residing in Chengdu, Sichuan Province (Sichuan Han) at an altitude of 500 m, 10 ethnic Han Chinese who migrated approximately 15 years ago from the plains to Lhasa (Tibet Han), and 12 native Tibetans who resided in Lhasa (Tibetan), a city in the Tibet Autonomous Region located at an altitude of 3,658 m. All of the participants were male and healthy, and written informed consent was obtained from all. Blood samples (~4 ml) were collected in tubes containing EDTA using a standard procedure. The study protocol was approved by the Ethics Committee of Chinese PLA General Hospital, in accordance with the Declaration of Helsinki.

Whole blood was centrifuged for 5 min at 2,500 rpm. The plasma and buffy coat layers were aspirated for initial removal of plasma, leukocytes and platelets. Mature RBCs were then passed through filter membranes for leukocyte removal and washed three times with phosphate-buffered saline (PBS) to obtain purified mature RBCs. We routinely assessed this material for residual platelet (PLT) and white blood cell (WBC) contamination using a hematology analyzer (BC-2800, MINDRAY, China). Typical readings indicating lack of contamination of these cells were approximately 0 WBCs/L and 0 PLTs/L. The purity of the resulting RBCs was verified by FACS for surface expression of CD235a (RBC, CD235a-FITC, Pharmingen, CA), CD45 (leukocyte, CD45-PE, Pharmingen, CA) and CD41a (platelet, CD41a-PE, Pharmingen, CA) using fluorescently labeled antibodies. We randomly selected purified RBCs for testing and the mean positive rates of CD235a, CD45 and CD41a were 98.1, 0.35, and 0.1%, respectively.

### RNA isolation and miRNA sequencing analysis

Total RNA was extracted from 100 μl purified RBCs of each sample using the TRIzol reagent (Invitrogen, CA, USA) according to the manufacturer's protocol. The Quantity and purity of the total RNA were measured using an RNA 6000 Nano Lab Chip Kit and Bioanalyzer 2100 (Agilent, CA, USA). Approximately 1 μg of total RNA was used to prepare a small RNA library according to the TruSeq Small RNA Sample Prep Kit protocol (Illumina, CA, USA), and single-end sequencing (36 bp) was performed on an Illumina HiSeq 2500 at LC-BIO (Hangzhou, China) according to the manufacturer's recommended guideline. All primary sequencing data have been deposited in the Gene Expression Omnibus (GEO dataset; GSE117436).

The raw reads were subjected to the Illumina pipeline filter (Solexa 0.3), and the dataset was further processed with an in-house program, ACGT101-miR (LC Sciences, Texas, USA), to remove adapter dimers, junk, low complexity molecules, common RNA families (rRNA, tRNA, snRNA, and snoRNA) and repeats. Unique sequences with 18–26 nucleotides in length were mapped to specific species precursors in miRBase 20.0 using a BLAST search to identify known miRNAs. miRNA differential expression based on normalized deep-sequencing counts was analyzed by selectively using Fisher's exact test, the Chi-squared 2X2 test, the Chi-squared nXn test, Student's *t*-test, and ANOVA depending on the experimental design. miRNAs with markedly altered expression levels between two groups were identified using fold change and *p*-value cutoffs of 2 and 0.05 for each test, respectively.

### Real-time PCR

Total RNA was extracted from RBCs using the miRcute miRNA isolation kit (TIANGEN, China) according to the manufacturer's instructions. RNA (1 μg) was reverse transcribed into cDNA using the miRcute Plus miRNA First-Strand cDNA Synthesis Kit (TIANGEN, China). Real-time PCR for miR-144-5p and miR-30b-5p was performed using the SYBR Green reagent (TIANGEN, China); U6 was used as the internal control. The relative amounts of miRNA or mRNA were determined using the comparative threshold cycle (CT) method.

### Hematology factor analysis

Hematological indices, including RBC count, hemoglobin concentration (HGB), hematocrit (HCT), mean corpuscular volume (MCV), mean corpuscular hemoglobin (MCH), mean corpuscular hemoglobin concentration (MCHC), WBC counts and PLT levels, were measured immediately after blood collection using commercial reagents with a BC-2800 analyzer (MINDRAY, China).

### Prediction of target genes of miRNAs

Two computational target prediction algorithms (TargetScan 50 and miRanda 3.3a) were used to identify miRNA binding sites to predict genes targeted by differentially expressed miRNAs. The data predicted by both algorithms were combined, and overlaps were calculated. GO (Gene Ontology) terms and KEGG (Kyoto Encyclopedia of Genes and Genomes) pathways of the differentially expressed miRNA targets were annotated.

### Data analysis

All analyses were performed using GraphPad Prism (version 5). Differences in variants among the groups were analyzed using one-way ANOVA or Student's *t*-test; *p* < 0.05 was considered statistically significant. Error bars indicate the standard deviation (SD). Data are expressed as the mean ± SD.

## Results

### Hematological features of participants

**Figure 1:**
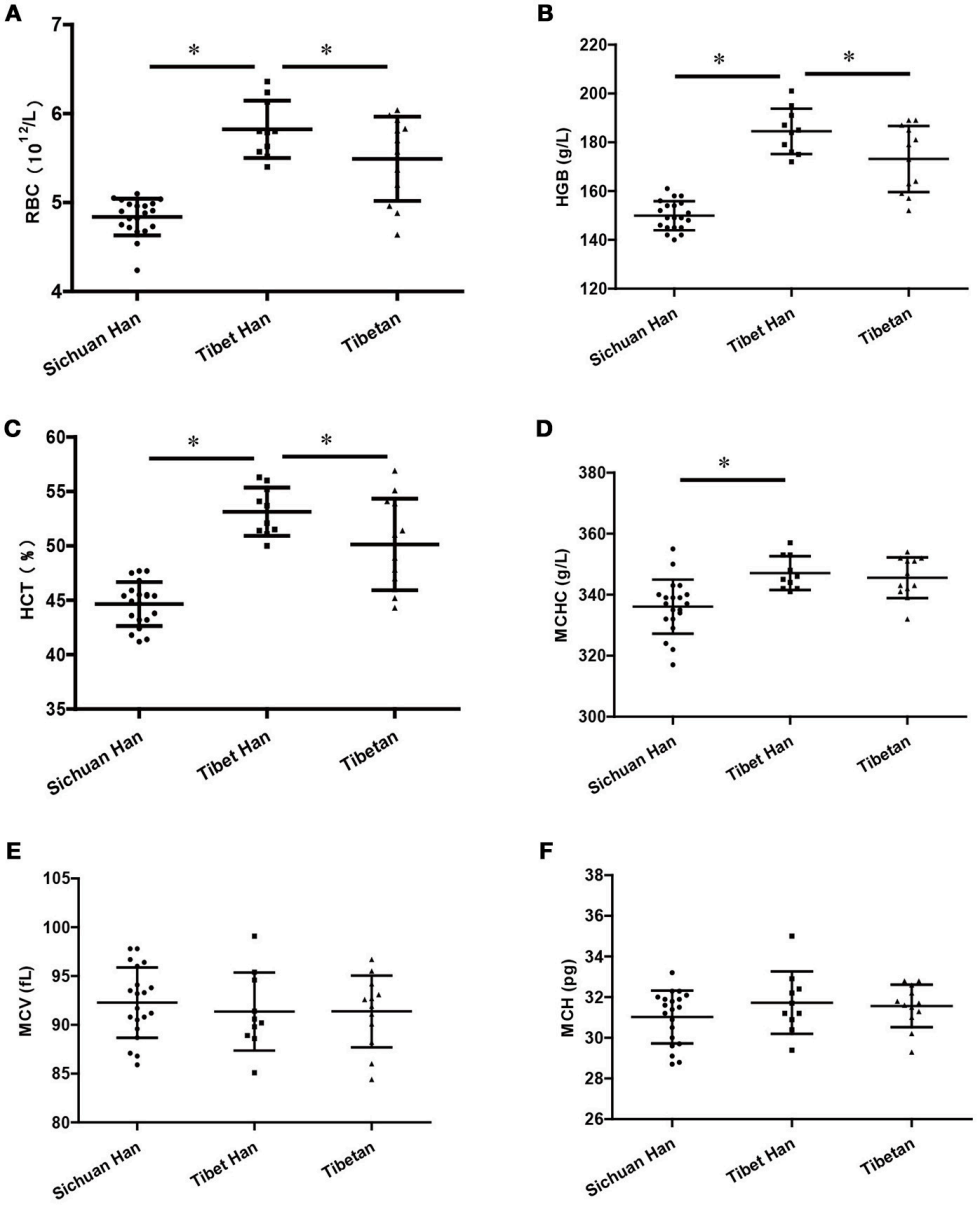
Hematological features of the Sichuan Han, Tibet Han and Tibetan groups. RBC, red blood cell counts **(A)**; HGB, hemoglobin **(B)**; HCT, hematocrit **(C)**; MCHC, mean corpuscular hemoglobin concentration **(D)**; MCV, mean corpuscular volume **(E)**; MCH, mean corpuscular hemoglobin **(F)** (*p-value < 0.05; error bars indicate the SD).

All participants in the Sichuan Han group, Tibet Han group, and Tibetan group were male; their ages were 24.3 ± 4.6 years, 40.1 ± 6.8 years, and 34.5 ± 12.3 years, respectively. The levels of RBCs (Figure 1A), HGB (Figure 1B), HCT (Figure 1C), and MCHC (Figure 1D) were significantly higher in the Tibet Han group than in the Sichuan Han group (*p* < 0.05). However, the values of RBCs, HGB and HCT were lower in the Tibetan group than in the Tibet Han group. No marked differences between the Tibet Han group and the Sichuan Han group were found for MCH (Figure 1E) and MCV (Figure 1F) values.

### miRNA sequencing analysis of RBC miRNA profiles

**Figure 2:**
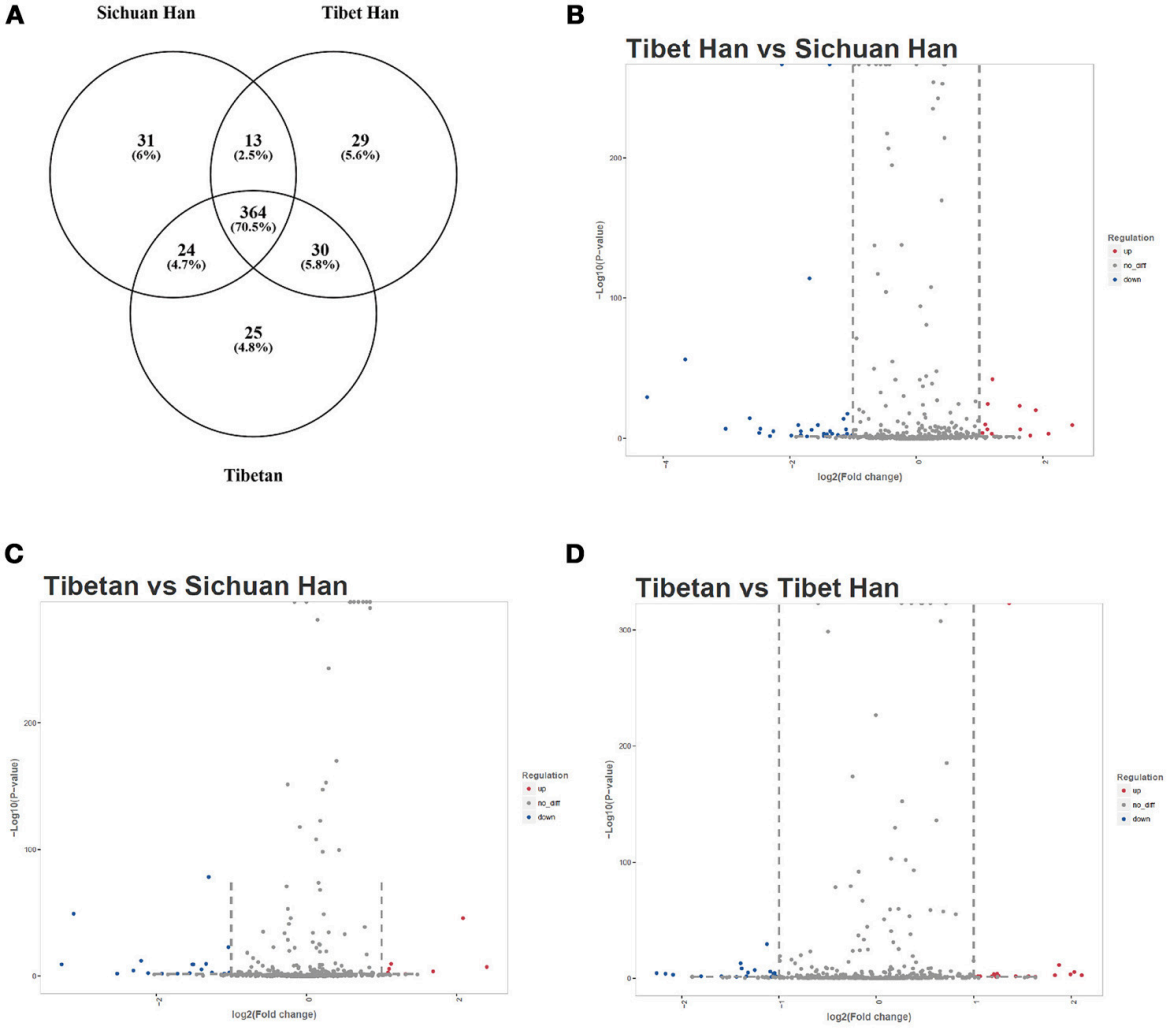
miRNA sequencing analysis of RBC miRNA profiles. miRNAs with more than 1 read in the sequencing data were considered expressed miRNAs. A Venn diagram **(A)** displays the overlapping miRNAs among the Sichuan Han group, Tibet Han group and Tibetan group. RBC miRNAs were compared between the Tibet Han group and the Sichuan Han group **(B)**, between the Tibetan group and the Sichuan Han group **(C)**, and between the Tibetan group and the Tibet Han group **(D)**. The volcano plot displays the relationship between fold change and significance using a scatter plot view. The red dots in the plot represent significantly upregulated miRNAs, and the blue dots indicate significantly downregulated miRNAs.

The RBC samples were separately pooled to produce three sample pools of the Sichuan Han, Tibet Han and Tibetan populations. We globally analyzed RBC miRNA expression profiles from the three RBC samples using miRNA sequencing. miRNAs with more than 1 read in the sequencing data were considered expressed miRNAs and we detected 516 miRNAs in the purified RBC samples (Supplementary Table 1), of which 364 overlapped among the three RBC groups. These miRNAs are shown in a Venn diagram (Figure 2A). Of the 516 miRNAs tested, 49 RBC miRNAs were differentially expressed (17 upregulated and 32 downregulated miRNAs) in the Tibet Han group compared with the Sichuan Han group (fold change > 2 or < 0.5) (Figure 2B), 33 RBC miRNAs were differentially expressed (12 upregulated and 21 downregulated miRNAs) in the Tibetan group compared with the Sichuan Han group (fold change > 2 or < 0.5) (Figure 2C), and 40 RBC miRNAs were differentially expressed (15 upregulated and 25 downregulated miRNAs) in the Tibetan group compared with the Tibet Han group (fold change > 2 or < 0.5) (Figure 2D). It is worth mentioning that among the differentially expressed miRNAs between the Tibet Han group and the Sichuan Han group, the downregulated miRNAs still exhibited considerably higher expression levels than the upregulated ones did. miR-144-5p, miR-30b-5p, miR-423-5p, miR-16-2-3p, miR-3200-5p, miR-4732-5p, miR-200c-3p, miR-125a-5p, miR-195-5p, and miR-141-3p were the top ten miRNAs with the highest levels of expression, and their fold changes were less than 0.5 between the Tibet Han group and the Sichuan Han group (Fisher's exact test, *p* < 0.001) (Table 1). The miRNA sequencing results demonstrated that in addition to markedly different RBC miRNA patterns in the Tibet Han group and the Tibetan group compared to the Sichuan Han group, significant differences in RBC miRNA profiles were also observed between the Tibetan Han group and the Tibetan group.

**Table 1:** Markedly altered miRNAs in pooled RBC samples from the Tibet Han group compared with the Sichuan group as determined by miRNA sequencing.

**miRNA**	**Sichuan Han**	**Tibet Han**	**Tibetan**	**Fold change**		
				**Tibet Han vs. Sichuan Han**	**Tibetan vs. Sichuan Han**	**Tibetan vs. Tibet Han**
miR-144-5p	32781.65	7520.45	19358.86	0.23^*^	0.59^*^	2.57^*^
miR-30b-5p	29093.81	13471.60	22569.93	0.46^*^	0.78^*^	1.68^*^
miR-423-5p	27877.61	10792.27	17083.94	0.39^*^	0.61^*^	1.58^*^
miR-16-2-3p	1429.75	444.64	579.33	0.31^*^	0.41^*^	1.30^*^
miR-3200-5p	425.78	200.68	315.54	0.47^*^	0.74^*^	1.57^*^
miR-4732-5p	306.88	138.70	235.80	0.45^*^	0.77^*^	1.70^*^
miR-200c-3p	283.51	22.63	33.08	0.08^*^	0.12^*^	1.46^#^
miR-125a-5p	123.97	42.30	43.26	0.34^*^	0.35^*^	1.02^#^
miR-195-5p	97.89	26.89	52.87	0.27^*^	0.54^*^	1.97^*^
miR-141-3p	97.55	15.74	21.21	0.16^*^	0.22^*^	1.35^#^

* *p-value < 0.001*,

# *p-value > 0.05, calculated by Fisher's exact test*.

### Verification of miRNA sequencing results by real-time PCR

**Figure 3:**
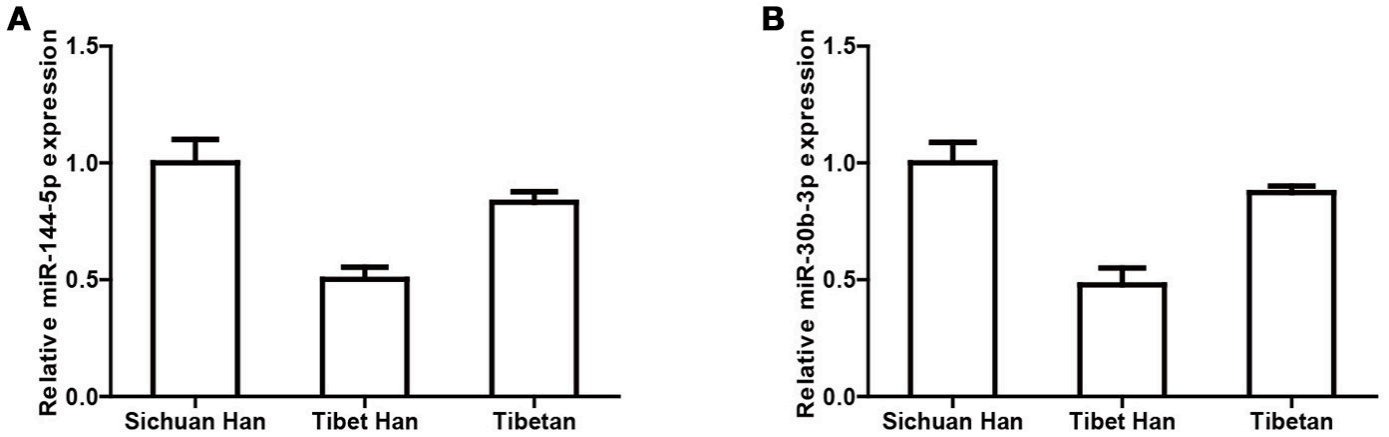
Verification of miRNA sequencing results by real-time PCR. Cq values of miR-144-5p **(A)** and miR-30b-5p **(B)** were converted to relative concentrations normalized to U6 values and were calculated using the comparative Cq method.

Next, to verify the miRNA sequencing results, we performed real-time PCR of miRNAs in individual RBC samples. We selected two miRNAs with the top two highest expression levels and considerable variation (miR-144-5p and miR-30b-5p) for real-time PCR validation of 5 randomly selected RBC samples from each group. U6 was used as the negative control. The variation trends in miR-144-5p (Figure 3A) and miR-30b-5p (Figure 3B) are consistent with the miRNA sequencing results.

### Target analysis of miRNAs altered by high-altitude hypoxia

**Figure 4:**
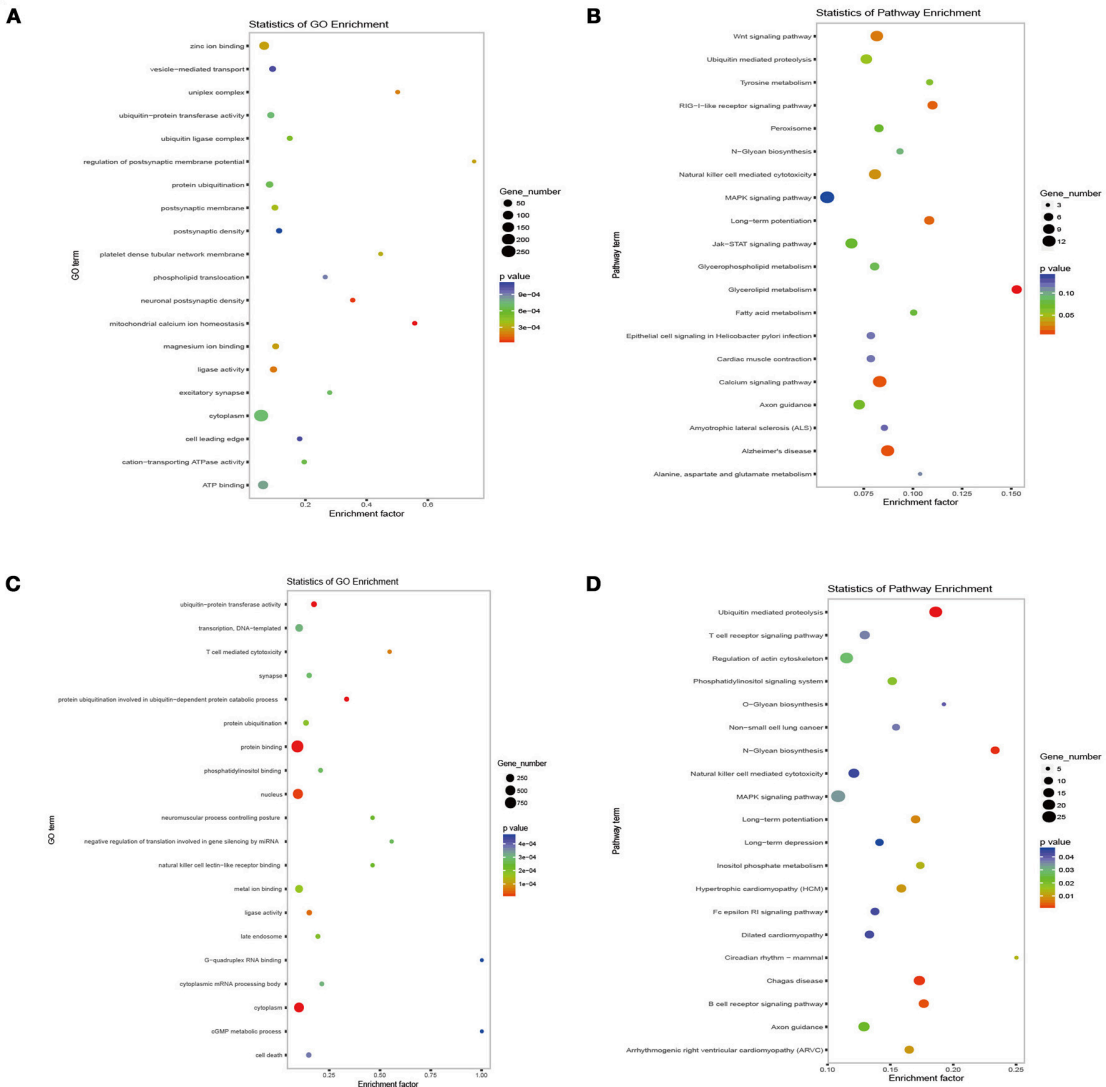
Target analysis of miRNAs altered by high-altitude hypoxia. The biological function prediction of miR-144-5p was performed by GO **(A)** and KEGG analysis **(B)**. The potential targets of miR-30b-5p by GO and KEGG analysis are shown in **(C,D)**.

To explore the possible roles and molecular basis of the miRNAs that were altered in response to high-altitude hypoxia, we performed a bioinformatic prediction of potential target genes of miR-144-5p and miR-30b-5p using two computational target prediction algorithms (TargetScan 50 and miRanda 3.3a). Based on GO terms and KEGG pathway analyses, the targets of miR-144-5p were found to be significantly associated with “ATPase activity” and “Calcium signaling pathway” (Figures 4A,B). Functional analysis of the potential targets of miR-30b-5p revealed a number of genes involved in “protein binding” and “regulation of actin skeleton” (Figures 4C,D). These results indicate that miR-144-5p and miR-30b-5p are involved in the regulation of cell energy and cell morphology. Further analysis demonstrated that the potential targets of both miRNAs are predicted to be associated with “hypoxia,” “erythroid,” and “NO”-related pathways (Table 2), Supplementary Table 2 exhibited a legend explaining the respective abbreviations of the target genes. Our findings suggest that miR-144-5p and miR-30b-5p may participate in adaptation to high-altitude hypoxic environments in humans.

**Table 2:** Predicted target genes of miR-144-5p and miR-30b-5p.

	**Hypoxia-related**			**Erythroid-related**			**NO-related**		
**miRNA**	**Target gene**			**Target gene**			**Target gene**		
miR-144-5p	PPARGC1A	SMAD4	MDM4	RAB14	EPB41L4B	PKNOX1	FOXO1	MAPK9	PTGS2
	NOX4	ITPR2		DMTN	L3MBTL3	PTBP3	IL10	RORA	
miR-30b-5p	UBE2D1	CUL2	IREB2	EPB41	ERG	ERBB4	MAPK9	RASD1	NEUROD1
	BECN1	PDGFA		EXOC6	PTPN2		GUCY1A3	CCL19	RORA

## Discussion

miRNAs are important epigenetic regulators of diverse cellular processes, and their dysregulation has been demonstrated in a range of bodily disorders or diseases (Lu et al., 2005; Ikeda et al., 2007). Recent studies have reported the effect of high-altitude hypoxia on human circulating miRNA profiles (Yan et al., 2015, 2016; Wang et al., 2017). It is known that, RBC counts in Han migrants increase markedly in response to the high-altitude hypoxic environment (Simonson et al., 2010), and abundant miRNAs can be found in mature RBCs (Chen et al., 2008; Kannan and Atreya, 2010; Wu et al., 2017). However, the effect of high-altitude hypoxia on RBC-derived miRNAs is still unclear. Here, we demonstrate that the RBC miRNA profiles of Han Chinese who migrated to highland Tibet over 10 years ago are dramatically different from those of Tibetans and those residing in low-altitude areas. These data suggest that human RBC miRNA patterns are significantly influenced by a high-altitude hypoxic environment.

In this study, only male samples were chosen for our analysis to reduce the number of confounding factors because Wu et al. reported that gender affects hemoglobin concentrations in Tibetans and Han Chinese (Wu et al., 2005). Several hematological indices were assessed, and RBC, HGB and HCT levels were notably higher in the Tibet Han group than in the Han group and the Tibetan group, which was consistent with previous reports (León-Velarde et al., 2000; Wu et al., 2005; Yan et al., 2015). A reasonable rise in RBC number and HGB levels is a beneficial adaptive response to high-altitude hypoxia, but their pathological increase can lead to elevated blood viscosity and aggravated tissue cell anoxia and result in high-altitude polycythemia accompanied by multiple organ dysfunction (Villafuerte and Corante, 2016). Therefore, the regulatory mechanisms of erythropoiesis in high-altitude environments need to be investigated thoroughly. Sufficient evidence proves that multiple miRNAs play key roles in the process of erythropoiesis (Felli et al., 2005; Sun et al., 2015; Li et al., 2017); thus, we sought to determine whether abundant RBC-derived miRNAs are involved in the regulation of erythropoiesis via transport pathways in high-altitude environments.

High-throughput miRNA sequencing was conducted, and the results showed that the expression profiles of RBC miRNAs of Tibet Han individuals were dramatically different from those of Sichuan Han individuals residing in the low-altitude area, with 49 differentially expressed miRNAs. Among them, miR-144-5p and miR-30b-5p, two miRNAs with the highest expression levels, were confirmed by real-time PCR in individual RBC samples. The consistent results of miRNA sequencing and real-time PCR indicated that RBC miR-144-5p and miR-30b-5p expression levels were significantly decreased in the Tibet Han group compared with the Sichuan Han group. Interestingly, the levels of both miRNAs in the Tibetan highlanders were also significantly lower than in the Han lowlanders, but higher than in the Tibet Han individuals. The expression trend of both miRNAs was negatively consistent with the variation trend of RBC and HGB levels, providing indirect evidence for the relationship between RBC miRNAs and erythropoiesis. In view of the stability of miRNA and its abundance in RBCs, further research on RBC-derived miRNAs may offer a new direction in the search for biomarkers of predicting adaptation to hypoxia. Subsequently, one question arises: Do erythrocyte-derived miRNAs function in high-altitude hypoxia, and if so, how?

To reveal the possible function and mechanism of RBC miRNAs, analysis of potential targets of miR-144-5p and miR-30b-5p was performed. GO and KEGG analyses indicated that the targets of both miRNAs are significantly associated with the “Calcium signaling pathway” and “Regulation of actin skeleton,” possibly indirectly affecting the formation and stability of RBCs. In addition, the potential target gene ERG of miR-30b-5p can regulate erythrocyte differentiation as an essential regulator of hematopoietic stem cell (HSC) function (Carmichael et al., 2012). Many studies indicate that miR-144 plays a crucial role in the process of erythropoiesis as an erythroid-specific regulator (Fu et al., 2009; Papapetrou et al., 2010; Rasmussen et al., 2010; Zhang et al., 2012; Undi et al., 2013). More encouragingly, biological experiments showed that miR-144 can increase the RBC number by inhibiting RAB14 transcription (Kim et al., 2015). Therefore, it is possible that RBC-derived miR-144-5p influences the process of erythropoiesis by targeting RAB14.

Many studies (Zhou et al., 2014; Thomou et al., 2017; Ying et al., 2017) confirm that extracellular miRNAs in microvesicles or exosomes may function in cell-cell communication. A similar transport mechanism of RBC miRNAs has been reported in Plasmodium infection research (Regev-Rudzki et al., 2013; Mantel et al., 2016; Wang et al., 2017). For example, Mantel et al. revealed that infected RBC-derived extracellular vesicles carrying the Ago2-miR-451 complex are taken up by endothelial cells and can alter vascular function in malaria. Wang et al. demonstrated that Ago2-miR-451 and miR-140 complexes in RBCs are abundantly released into parasites within infected RBCs, targeting the mRNAs of *P. falciparum* erythrocyte membrane protein-1 (pfEMP1, a critical parasite antigen). In addition, Kumar et al. demonstrated that bone marrow HSCs can take up exosomes derived from acute myeloid leukemia, and normal hematopoiesis is suppressed (Kumar et al., 2018). Papapetrou et al. observed that the expression level of miR-144 increases during erythropoiesis in HSCs and miR-144 can positively regulate erythrocyte differentiation (Papapetrou et al., 2010). Therefore, we speculated that miR-144-5p derived from erythrocyte vesicles may be taken up by the HSCs and inhibit the expression levels of target genes (e.g., RAB14), resulting in promotion of erythropoiesis.

The selective mechanism of miRNA loading into exosomes can be modulated by pathophysiological changes (Skog et al., 2008; Guduric-Fuchs et al., 2012; Bhome et al., 2018). Therefore, we infer that the amounts of miR-144-5p released by microvesicles might be affected by hypoxia and other factors; that is, the RBC vesicles released by the Tibet Han population may carry higher amounts of miR-144-5p to HSCs to promote erythropoiesis than the Sichuan Han population, resulting in significantly reduced miR-144-5p expression levels in RBCs, correspondingly, the RBCs of the Tibetan group release lower amounts of miR-144-5p than the Tibet Han group, which may partly contribute to a lower level of RBC production and prevent excessive RBC increase in Tibetans. However, further testing of this hypothesis should be conducted, to obtain more direct evidence supporting the physiological role of erythrocyte-derived miRNAs in hypoxia.

Furthermore, the expression level of PPAR (Peroxisome Proliferator Activated Receptor) family, as a potential target gene of miR-144-5p, is repressed under hypoxia (Slot et al., 2014) and negatively correlated with HGB concentration (Simonson et al., 2010), implying that miR-144-5p has the potential to regulate a high-altitude adaptation. In addition, potential target genes of miR-144-5p and miR-30b-5p, such as FOXO1 and MAPK9, are also associated with the NO synthesis-related signaling pathway, which can affect blood flow and the oxygen carrying capacity of individuals in response to hypoxia. Nonetheless, the targets are less specific for NO, meaning this may not be the main function of both miRNAs.

## Conclusions

Collectively, we investigated for the first time that a high-altitude hypoxic environment has marked effects on human RBC miRNA expression profiles. By bioinformatic analyses, some potential target genes of affected miRNAs (miR-144-5p and miR-30b-5p) exhibited enrichment in the erythroid-, hypoxia- and NO- related signaling pathways in response to hypoxia. Further investigation of more individuals, including females and individuals of other ages, should be performed to confirm our results. Moreover, further studies are needed to clarify the potential of RBC miRNAs as biomarkers for predicting high-altitude hypoxic adaptation and the possible physiological role of miR-144-5p, miR-30b-5p and other RBC-derived miRNAs, the levels of which are altered under high-altitude hypoxic conditions.

## Author contributions

DW and CL conceived and designed the experiments. LS performed the experiments. FF contributed to sample and physical data collections. RL and BN analyzed part of the data. LZ, SY, and SW assisted in part of the experiments. LS wrote the manuscript.

### Conflict of interest statement

The authors declare that the research was conducted in the absence of any commercial or financial relationships that could be construed as a potential conflict of interest.

## Supplementary material

Supplementary Table 1 Differentially expressed miRNAs in purified RBC samples from the Sichuan Han, Tibet Han and Tibetan groups determined by high-throughput miRNA sequencing.

Supplementary Table 2 Explanation for the abbreviation of the target genes showed in Table 2.
